# Development of a two-step nucleic acid amplification test for accurate diagnosis of the *Mycobacterium tuberculosis* complex

**DOI:** 10.1038/s41598-021-85160-2

**Published:** 2021-03-11

**Authors:** Chien-Ru Lin, Hsin-Yao Wang, Ting-Wei Lin, Jang-Jih Lu, Jason Chia-Hsun Hsieh, Min-Hsien Wu

**Affiliations:** 1 Graduate Institute of Biomedical Engineering, Chang Gung University, Taoyuan, Taiwan; 2 Department of Laboratory Medicine, Chang Gung Memorial Hospital at Linkou, Taoyuan City, Taiwan; 3 Ph.D. Program in Biomedical Engineering, Chang Gung University, Taoyuan City, Taiwan; 4 Division of Haematology/Oncology, Department of Internal Medicine, Chang Gung Memorial Hospital at Linkou, Taoyuan City, Taiwan; 5 Division of Haematology/Oncology, Department of Internal Medicine, New Taipei Municipal Hospital, New Taipei City, Taiwan; 6 School of Medicine, Chang Gung University, Taoyuan City, Taiwan; 7 Department of Medical Biotechnology and Laboratory Science, Chang Gung University, Taoyuan City, Taiwan; 8 Department of Chemical Engineering, Ming Chi University of Technology, New Taipei City, Taiwan

**Keywords:** Microbiology, Molecular biology, Health care

## Abstract

The *Mycobacterium tuberculosis* complex (MTBC) remains one of the top 10 leading causes of death globally. The early diagnosis of MTBC can reduce mortality and mitigate disease transmission. However, current nucleic acid amplification diagnostic test methods are generally time-consuming and show suboptimal diagnostic performance, especially in extrapulmonary MTBC samples or acid-fast stain (AFS)-negative cases. Thus, development of an accurate assay for the diagnosis of MTBC is necessary, particularly under the above mentioned conditions. In this study, a single-tube nested real-time PCR assay (N-RTP) was developed and compared with a newly in-house-developed high-sensitivity real-time PCR assay (HS-RTP) using 134 clinical specimens (including 73 pulmonary and 61 extrapulmonary specimens). The amplification efficiency of HS-RTP and N-RTP was 99.8% and 100.7%, respectively. The sensitivity and specificity of HS-RTP and N-RTP for the diagnosis of MTBC in these specimens were 97.5% (77/79) versus 94.9% (75/79) and 80.0% (44/55) versus 89.1% (49/55), respectively. The sensitivity and specificity of HS-RTP and N-RTP for the diagnosis of MTBC in pulmonary specimens were 96.3% (52/54) versus 96.3% (52/54) and 73.7.0% (14/19) versus 89.5% (17/19), respectively; in extrapulmonary specimens, the sensitivity and specificity of HS-RTP and N-RTP were 100% (25/25) versus 92% (23/25) and 83.3% (30/36) versus 88.9% (32/36), respectively. Among the AFS-negative cases, the sensitivity and specificity of HS-RTP and N-RTP were 97.0% (32/33) versus 90.9% (30/33) and 88.0% (44/50) versus 92.0% (46/50), respectively. Overall, the sensitivity of HS-RTP was higher than that of N-RTP, and the performance was not compromised in extrapulmonary specimens and under AFS-negative conditions. In contrast, the specificity of the N-RTP assay was higher than that of the HS-RTP assay in all types of specimens. In conclusion, the HS-RTP assay would be useful for screening patients suspected of exhibiting an MTBC infection due to its higher sensitivity, while the N-RTP assay could be used for confirmation because of its higher specificity. Our results provide a two-step method (screen to confirm) that simultaneously achieves high sensitivity and specificity in the diagnosis of MTBC.

## Introduction

The *Mycobacterium tuberculosis* complex (MTBC) is responsible for one of the most important infectious diseases worldwide, causing 1.5 million deaths annually^1^. According to government statistics, although the incidence rate of MTBC infection showed a steadily decreasing trend from 2005–2018, in Taiwan, there were still 38.9 cases and 2.1 MTBC-related death cases per 100,000 population in 2018^2^. To reduce the morbidity and mortality of MTBC infection and prevent its transmission, an accurate and rapid diagnosis in the early stage is particularly important^3^.

The conventional approach for MTBC diagnosis relies primarily on the microscopic detection of acid-fast bacilli (AFB) in smears^4^, followed by MTBC culture with selective medium^4,5^. Although acid-fast staining (AFS) is a rapid and simple procedure, the sensitivity of MTBC diagnosis is 71.4% in pulmonary specimens and drops to 24% in extrapulmonary specimens^6^. This compromised performance results from the paucibacillary nature of extrapulmonary specimens and the fact that inhibitors are more common in extrapulmonary specimens than in pulmonary specimens (approximately fivefold)^7^. In addition, the AFS method cannot distinguish between MTBC and nontuberculosis mycobacteria (NTM)^8^. Currently, mycobacterium culture is regarded as the gold standard method for MTBC diagnosis. Although mycobacterium culture shows a high detection specificity, its sensitivity is low (39–80%), mainly resulted from the slow-growing nature of mycobacteria^9,10^. Compared with the AFS method, however, the major drawback of mycobacterium culture is that it is time-consuming.

To improve the diagnosis of MTBC, various nucleic acid amplification tests (NAATs) have been developed over the past few decades^11–14^. In general, the detection sensitivity and specificity of NAATs ranges from 64–100% and 74–99.3%, respectively; however, this performance varies from 40–84% in paucibacillary specimens. Various NAATs show sensitivity ranging from 95 ~ 100% in AFS-positive specimens, while the sensitivity drops to 40 ~ 60% in AFS-negative pulmonary specimens^15^. In addition, some reports have indicated that there are several inhibitors in sputum that might affect nucleic acid amplification and cause false-negative NAAT results, and this phenomenon mostly occurs in AFS-negative specimens^14,16^. Moreover, sporadic or systematic errors (e.g., a primer/probe exhibiting cross-reactivity to NTM or other species of bacteria, viruses or fungi) can cause false positivity of NAAT results^17^. Taken together, the above findings highlight the importance of developing a highly sensitive and specific NAAT method capable of detecting MTBC in AFS-negative specimens and extrapulmonary specimens.

Most NAATs are based on the detection of multicopy insertion sequences (ISs), which is expected to increase the sensitivity of the tests. IS986, IS987, IS1081 and IS6110 have long been used as NAAT targets for the diagnosis of MTBC^18–21^. In nature, ISs, which exhibit high transposition ability, play a role in the major regions of bacterial repetitive elements; these sequences are therefore often used for typing different species and strains and can help their host adapt to the environment^22^. In the application of NAATs, ISs, especially IS6110, have been used in multiplex PCR for the diagnosis of MTBC in various types of clinical specimens, including pulmonary and extrapulmonary specimens, as such sequences exhibit high copy numbers in most MTBC strains (up to 25 copies per genome)^23^, although strains with only a single copy or no copies have also been identified in rare cases, as found in *M*. *bovis* and its substrains, such as *M*. *bovis* BCG^24^. The commercial Xpert MTB/RIF Ultra kit (Sunnyvale, USA, Ultra), which targets IS6110 and IS1081 for detection, is recommended by the WHO as the initial diagnostic test for all adults and children with signs and symptoms of MTBC^25^. It is one of the most widely used automated, integrated, cartridge*-*based molecular assay systems for NAATs; it showed 87.5% sensitivity (95% confidence interval [CI], 82.1% to 91.7%) among all specimens, and the limit of detection (LOD) of MTB/RIF Ultra was 15.6 bacterial colony-forming units per ml^26^. In pulmonary specimens, the sensitivity and specificity of the Ultra assay were 88% (CI: 85% to 91%) and 96% (CI: 94% to 97%), respectively^25^. For extrapulmonary specimens, the sensitivity and specificity were 98.5% and 97%, respectively; however, the assay showed significantly decreased sensitivity [78.9% (CI: 70.0% to 86.1%)] in sputum AFS-negative samples^27^. In addition, the Ultra assay is costly (the price of the cartridge is $9.98), and it is difficult to perform wide testing in many low- and middle-income countries with this test^28^. Thus, in-house-developed real-time PCR tests are widely used in developing countries because these tests are less expensive than commercial kits.

Nested-PCR assays based on NAAT mainly involve the use of two primer sets and a two-step procedure, with amplification by the external primers first, followed by inner primers. Although reports in the literature have demonstrated that the use of nested PCR for the rapid diagnosis of extrapulmonary MTBC can improve the performance of detection in terms of both sensitivity and specificity^29–31^, some reports have shown that the performance of nested PCR for the diagnosis of extrapulmonary specimens is directly related to the different sample types assessed, even when they are collected from the same case, with sensitivity of 72.2% reported in blood and/or urine, but a dramatic drop to 33.3% in pleural fluid specimens^32^. Some other technical shortcomings of nested PCR include the complicated and, thus, time-consuming procedures required for the two-step operation, increasing the risk of cross-contamination during the process, and its high cost. To address these issues, the technique of single-tube or single-step nested PCR was proposed. In practice, the outer primers are designed to present a higher annealing temperature than the inner primers. The reaction is initiated at a high annealing temperature for a few cycles, followed by decreasing to a lower temperature to allow the inner primer to bind and for amplification. A previous study showed that the overall sensitivity of single-tube nested PCR was 89% for pulmonary specimens and 42% for extrapulmonary specimens. More recently, single-tube nested real-time PCR has been reported to show improved MTBC detection performance [i.e., sensitivity (97.2%), specificity (99.7%)], and the LOD is 5 colony-forming unit per ml^33^ in overall respiratory specimens. Choi et al. reported that sensitivity of simple IS6110 real-time PCR and single-tube nested real-time PCR was 94.6% (158/167) and 100% (167/167) for sputum specimens, respectively^34^. Nevertheless, the detection sensitivity of this approach in AFS-negative or extrapulmonary specimens is still a problem. This highlights the importance of developing a new nested PCR method for the diagnosis of MTBC in extrapulmonary and AFS-negative specimens.

In our previous study, we developed, optimized, and quantified the sensitivity and specificity of the detection of MTBC using IS4 primer/probe pairs; however, we were not satisfied with the specificity of that assay due to minor cross-reactivity for NTM^35^. Here, we report two newly developed MTBC assay methods: an in-house-developed high-sensitivity real-time PCR (HS-RTP) assay and a single-tube nested real-time PCR assay (N-RTP). The results demonstrate that the N-RTP assay can increase specificity from 80% to 89.1% compared with HS-RTP, while the sensitivity of HS-RTP (97.5%) is slightly higher than that of N-RTP (94.5%). Because of the high sensitivity of the HS-RTP approach, the diagnosis of MTBC could first be approached by HS-RTP screening and then be confirmed by N-RTP abased on its higher specificity. We concluded that the two-step assay method (screening followed by confirmation) achieved a higher sensitivity and specificity in the detection of MTBC and that its performance was not compromised in extrapulmonary specimens or under AFS-negative conditions.

### Methods

### Multiple nucleotide sequence alignment of IS6110 and IS1081 fragments in 8 *Mycobacterium tuberculosis* complexes (MTBCs)

The alignment results for IS6110 were previously described^35^. The mycobacterial strains were identified in a BLAST search of the National Center for Biotechnology Information database (http://www.ncbi.nlm.nih.gov/BLAST/). The strains included the *M. africanum* strain 25, *M. bovis* BCG str. Tokyo172, *M. caprae* strain Allgaeu, *M. microti* strain 12, *M. canettii* CIPT 140010059, *M. bovis* AF2122/97, *M. tuberculosis* H37Ra, and *M. tuberculosis* H37Rv. The IS1081 nucleotide sequences were aligned with Vector NTI 9.0 software (Invitrogen, Carlsbad, CA).

### Design of primers and probes

The primers and probes were selected to target the IS6110 and IS1081 sequences of the conserved region of MTBC using the online PrimerQuest Tool from the IDT website (https://sg.idtdna.com/PrimerQuest/Home/Index), which were compared to all the available sequences with BLAST (http://www.ncbi.nlm.nih.gov/BLAST/). The IS6110-specific primer and probe set (IS4F and IS4R) was designed as previously described. As an internal control, a primer and probe for bacteriophage lambda (cI857ind 1 Sam 7) were designed to amplify a 111 bp amplicon within the 35,000–36,000 region, for which there is no conserved sequence in the BL21(DE3) *E. coli* strain, and were designated Ld2F and Ld2R, respectively. The fluorogenic probes IS4P, 1081P, and Ld2P were labeled with a unique fluorescent reporter dye [6-carboxyfluorescein (FAM)], [4,4,7,2′,4′,5′,7′-hexachloro-6-carboxyfluorescein (HEX)], and [indocarbocyanine (Cy5)] at the 5′-end, respectively, and an internal ZEN Quencher and a 3′-Iowa Black Fluorescent Quencher (IBFQ) at the 3′-end.

### Real-time assay and cycling conditions

The HS-RTP and N-RTP PCR assays were performed using the TaqMan system (CFX96 TouchTM Real-Time PCR Detection System, Bio-Rad Laboratories, Inc., USA). Each real-time PCR assay was carried out in a 13.2 µl reaction including 1 × QPCR master mix (TOYOBO, Japan), 0.76 µM external primers (Nes6110F, Nes6110R, Nes1081F and Nes1081R), 0.15 µM internal primers (IS4F, IS4R, 1081F, 1081R), the IS6110 probe (IS4P) at 0.15 µM, the IS1081 probe (1081P) at 0.15 µM, 0.3 µM internal control primers (Ld2F and Ld2R), the internal control probe (LdP) at 0.15 µM, 600 copies of Lambda plasmid DNA (internal control), and 4 µl of DNA extracted from the specimen. The one-step qPCR assay was separated into two amplification stages, which were carried out under the following conditions: the first amplification stage involved initial denaturation at 95 °C for 2 min, followed by 9 cycles of 95 °C for 5 s and 75 °C for 10 s, and the second amplification stage involved 45 cycles of denaturation at 95 °C for 5 s and annealing/extension at 60 °C for 10 s. The fluorescence emission signal was detected at the end of every run during the second amplification stage. The cycle threshold (Ct) values were automatically determined in a CFX96 system (Biorad) in multivariate, nonlinear regression mode.

### Clinical specimens

The authors declare that this study was approved by the Chang Gung Medical Foundation Institutional Review Board (IRB no. 201901791B0) and granted a waiver of patient consent. All methods were performed in accordance with the relevant guidelines and regulations. A total of 134 specimens were collected from the Department of Laboratory Medicine of Chang Gung Memorial Hospital at Linkou between Feb 2019 and Feb 2020. The clinical specimens requested for MTBC PCR testing (Cobas TaqMan MTB assay (CTM, Roche)) were obtained from patients who were suspected of having TB, as judged by clinical physicians and depending on the patients’ clinical signs and symptoms. The pulmonary samples included sputum (SP), bronchial wash (BW), and bronchoalveolar lavage fluid (BAL) specimens, while the extrapulmonary samples consisted of tissue (TS), cerebrospinal fluid (CSF), pleural effusions (PL), pus, ascites (AS), fresh tissue (FTS), synovial fluid (SY), and other types (OTH) (data were missed in our database). All the specimens were collected according to the clinical purposes of daily practice rather than being specifically obtained for this study. Among these samples, 73/134 (54.5%) and 61/134 (45.5%) samples were collected from pulmonary and extrapulmonary specimens, respectively, while 51/134 (38.1%) and 83/134 (61.9%) specimens were classified as belonging to the AFS-positive and AFS-negative groups, respectively. The frequency of clinical specimens according to the type of sample is shown in Table 1.

**Table 1:** Frequency of clinical specimens on the type of sample in the study.

Nature of specimen	Acid-fast stain		Total
	Positive	Negative	
**Pulmonary specimens, n = 73 (54.5%)**			
Sputum	36	7	43
Bronchoalveolar lavage (BAL)	3	18	21
Bronchial washing (BW)	4	5	9
**Extra-pulmonary specimens, n = 61 (45.5%)**			
Tissue (TS)	4	26	30
Cerebrospinal fluid (CSF)	11	0	11
Pleural effusion (PL)	10	0	10
Pus	4	1	5
Other (OTH)^a^	0	3	3
Ascites (AS)	0	1	1
Synovial fluid (SY)	0	1	1

^a^Data were not actualized for various reasons.

## Results

### Primers and probes designed for HS-RTP and N-RTP

**Figure 1:**
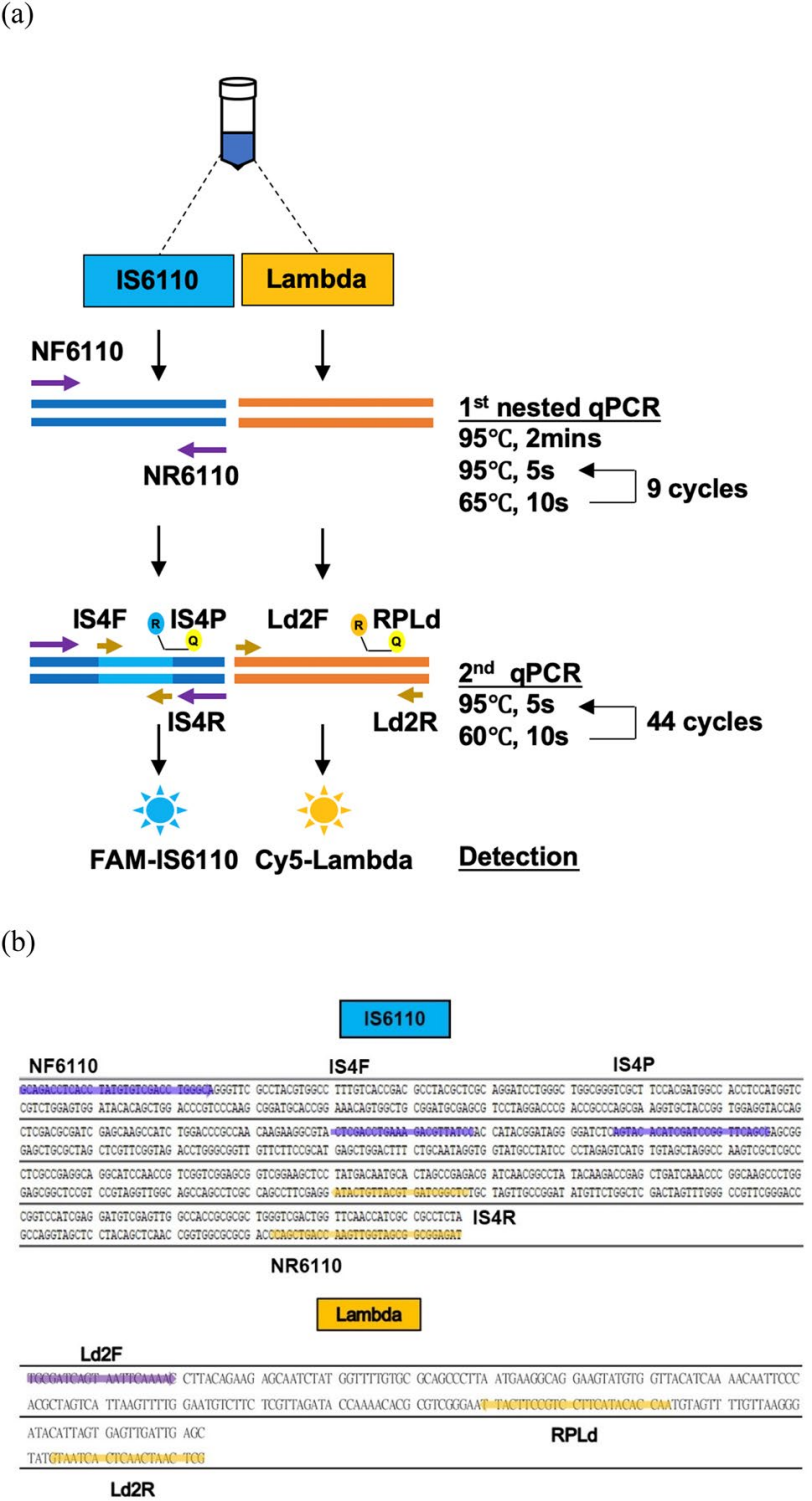
Schematic representation of the single-tube nested real-time PCR amplification and sequences of primers/probes. (**a**) Schematic showing how the single-tube nested qPCR works. At the first round, the PCR was performed to amplify a 428-bp fragment with an external primer set (NF6110 and NR6110). The larger amplicon produced by the first round of PCR was used as a template for the second round of PCR. The second pair of primers and probe (IS4F, IS4R and IS4P) bind within the first round of amplicon to produce a fragment shorter in length (141-bp). Second round amplicons were detection with a FAM-labeled probe (495–520 nm). The internal control (Lambda phage DNA) is only co-amplified with target DNA (IS6110-containing vector) with Ld2F, Ld2R and RPLd at the second round of PCR and the products were detection with a Cy5-labeled probe (646-662 nm). The PCR program of the assay was shown in the right-hand side. (**b**) The nested-qPCR amplicon of *IS6110* from MTBC and *Lambda* nucleotide sequences are shown. The sense primers were shown as black letters on a purple background and the anti-sense primers were indicated as black letters on an orange background.

Previously, we successfully developed IS4 primer/probe sets for the detection of MTBC. Here, we designed an outer primer pair, Nes6110, that covers the IS4 amplicon and amplifies a larger fragment in the first-round amplification reaction. The amplicons produced by Nes6110 in the first round of PCR are used as the DNA template for the secondary amplification step for IS4. In addition to designing Nes6110, we designed a new primer/probe set for Lambda plasmid DNA, Ld2, which was used as an internal control in this study. The Nes6110 primer was designed with a higher melting temperature (Tm) = 65 °C than those for IS4 and Ld2 (Tm = 60 °C for both). The amplicons of IS4 and Ld2 were designed to exhibit different fluorescence excitation and emission wavelengths, in which the 6-FAM-labeled IS4P probe emits blue fluorescence, and the Cy5-labeled Lambda DNA probe (RPLd) emits red fluorescence. In the preliminary analyses, we tested over 6 primer/probe sets to eliminate cross-reactivity between IS4 and the newly designed primer/probe pairs (data not shown). While Ld2 and Nes6110 elicited interference from IS4, the effect was minor. In practice, of HS-RTP, IS4 and Ld2 were used for the amplification of IS6110 and Lambda DNA. The amplification stage involved initial denaturation at 95 °C for 2 min, followed by 45 cycles of denaturation at 95 °C for 5 s and annealing/extension at 60 °C for 10 s. In practice, the first amplification stage of N-RTP (for Nes6110 amplification) involved initial denaturation at 95 °C for 2 min, followed by 10 cycles of 95 °C for 5 s and 65 °C for 10 s, and the second amplification stage involved 45 cycles of denaturation at 95 °C for 5 s, after which the temperature was decreased to 60 °C for 10 s for annealing/extension to allow IS4 and Ld2 binding and amplification (Fig. 1a,b). In summary, we successfully established primer/probe pairs for N-RTP (with Nes6110) and HS-RTP (without Nes6110) assays.

### Validation of the HS-RTP and N-RTP assays with internal standards revealed efficient amplification and increased sensitivity

**Figure 2:**
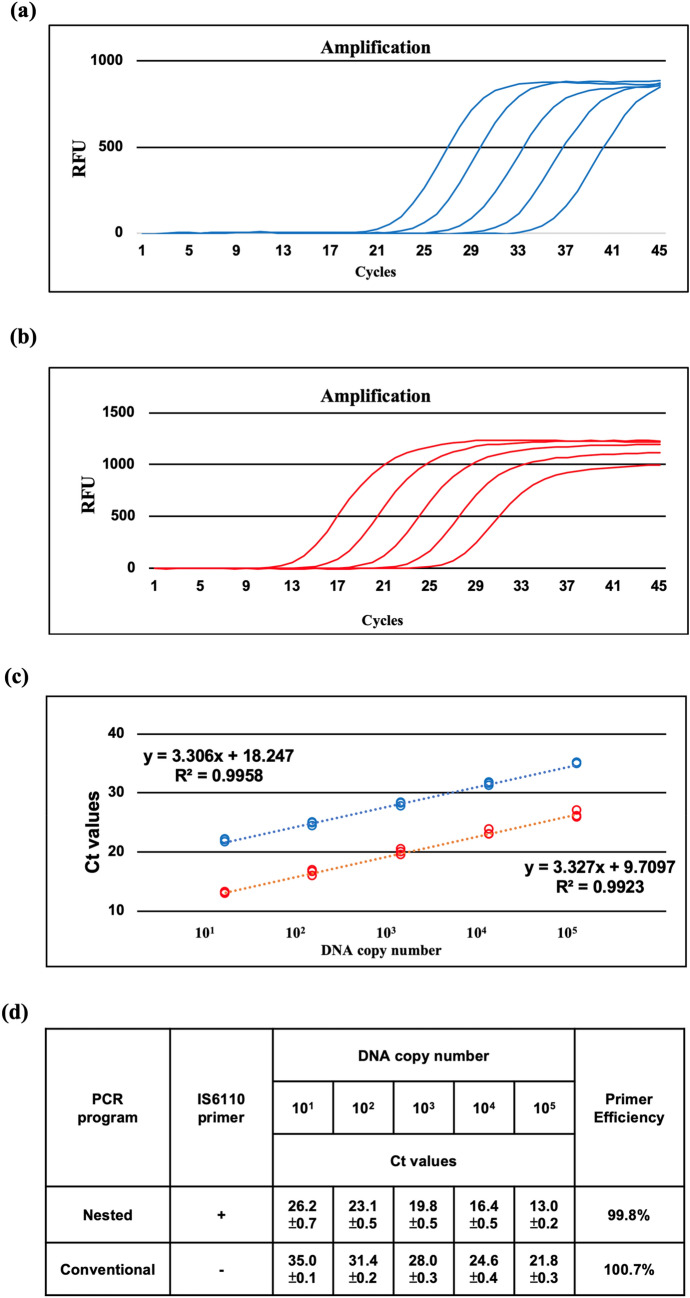
Single-tube nested real-time PCR assay using five samples of which Lambda DNA (1000 copies) was mixed with tenfold serial dilutions of *IS6110*-containing synthetic vectors from 10^5^–10^1^ DNA copies. Amplification curve of conventional real-time PCR (**a**) and single-tube nested real-time PCR (**b**). Detection of *IS6110* target sequence with a FAM-labeled double quenched probe (495–520 nm) showing the increasing number of cycles required to detect reducing DNA copies. The relative fluorescence units (y axis) of the reaction is plotted against the Ct values (x axis). The amplification curves are representative of three independent experiments. (**c**) Standard curve of single-tube nested real-time PCR (red circles) and conventional PCR (blue circles). The Ct values (y axis) are plotted against the log of the starting quantity of *IS6110*-containing synthetic vectors DNA copies (x axis) for each dilution. There are representatives of three independent experiments in each dilution. The slope, Ct values, Y-intercept, and R^2^ are shown. (**d**) The Ct values and primer efficiency with and without nested PCR are shown.

To verify the performance of HS-RTP and N-RTP amplification, primer and probe set sensitivity was investigated. Synthetic IS6110-containing plasmid DNA targeted by the IS4 primer/probe set was obtained, which was serially diluted tenfold from 10^5^ to 10^1^ genomic DNA copies, mixed with bacteriophage lambda DNA (equal to 10^3^ genomic DNA copies), and used to produce a standard curve. A comparison of HS-RTP and N-RTP showed that the fluorescence resulting from N-RTP was higher than that from HS-RTP (Fig. 2a,b), and the standard curve showed average slope values in this assay (-3.31 for HS-RTP, − 3.32 for N-RTP). The primer amplification efficiency of qPCR was 100.7% in the HS-RTP assay and 99.8% in the N-RTP assay, based on the equation AE = [10^−1/slope^ − 1]. The overall results showed a similar correlation coefficient between HS-RTP and N-RTP (Fig. 2c). Compared with HS-RTP (Ct values from 21.8 to 35.0), the N-RTP results produced amplification curves with relatively lower Ct values (13.0 to 26.2), meaning that effective amplification in the N-RTP assay may increase sensitivity (Fig. 2d). In the study, we used Lambda DNA as the internal control. Specifically, we added synthetic vectors containing Lambda DNA as the internal control. Given that the Lambda DNA primer/probe sets may interrupt primer efficiency of HS-RTP and N-RTP assays, we spiked lower copies of Lambda DNA and decreased the primer/probe concentration. Our tests suggested that 30 cycles are required to detect 1,000 copies of Lambda DNA (data not shown).

### The N-RTP assay LOD was estimated to be a single copy

IS6110-containing plasmid DNA with five gene copies or a single gene copy was used to determine the LOD of the assay, and all tests were performed in twenty replicates^36^. The N-RTP assay revealed a 100% positive rate, while the HS-RTP assay showed lower positive rates (100% for five copies and 95% for a single copy) (Table 2). The detection limit of N-RTP is estimated to be a single copy and one to five copies for HS-RTP.

**Table 2:** Limit of detection of *IS6110*-qPCR assay.

DNA copy number	N-RTP (with all primer)^b^		HS-RTP^a^ (without external primer)^c^	
	Average Ct values (± SD)	Interpretation (representation)	Average Ct values (± SD)	Interpretation (representation)
5	27.5 ± 0.6	Positive (20/20)	36.5 ± 0.3	Positive (20/20)
1	30.2 ± 0.8	Positive (17/20)	39.2 ± 0.9	Positive (19/20)
0	ND	Negative	ND	Negative

Ct, Cycle threshold; SD, Standard Deviation; ND, not determined.

^a^High sensitive real-time PCR condition were described by Wang et al. 2019.

^b^All primers contain NF6110, NR6110, IS4F, IS4R, IS4P, Ld2F, Ld2R, RPLd.

^c^External primers (NF6110 and NR6110) were replaced by TE buffer.

### Analytical specificity

To check for cross-reactivity, eleven NTM species, six bacterial species, and nine fungal species were evaluated in the HS-RTP and N-RTP assays. Finally, no amplification was observed with any of the primers when genomic DNA was extracted from individual species, and the input was 10^6^ copies. All the strains were obtained from the microbiology laboratory of Chang Gung Memorial Hospital (CGMH) in Linkou (Supplemental Table 1).

### Comparison of the HS-RTP and N-RTP with a standard NAAT and mycobacterium culture

There were 134 clinical specimens subjected to testing by the HS-RTP and N-RTP assays. Cobas TaqMan MTB (CTM) qPCR assay (Roche) was used as the standard NAAT. Mycobaterium culture was used as the referral standard. Among 79 culture-positive specimens, the HS-RTP and N-RTP assays yielded positive results in 77 (97.5%) and 74 (93.7%) samples, respectively (*P* > 0.05). Additionally, among 55 culture-negative specimens, the HS-RTP and N-RTP assays revealed negative results in 44 (80%) and 49 (89.1%) specimens (*P* > 0.05), respectively, meaning that more false-positive specimens (five specimens) were obtained by N-RTP (Table 3, panel: All specimens). In both pulmonary and AFS-positive specimens, the results were similar between HS-RTP and N-RTP for culture-positive specimens, while the N-RTP assay elicited three additional false-positive results from nineteen and five of these specimens, respectively (Table 3, panel: pulmonary and AFS-positive specimens). Additionally, the HS-RTP assay showed 25 and 32 positive results among 25 and 33 extrapulmonary and AFS-negative specimens that were not detected as positive by the N-RTP assay in culture-positive specimens. Among 36 and 50 culture-negative extrapulmonary and AFS-negative specimens, respectively, there were an additional 2 specimens identified as positive by the N-RTP assay (Table 3, panel: extrapulmonary and AFS-negative specimens).

**Table 3:** Performance comparisons between the HS-RTP, the N-RTP, and culture under different conditions, including overall specimens, pulmonary specimens (sputum, bronchoalveolar lavage (BAL) and bronchial washing (BW)), extrapulmonary specimens, AFS-positive specimens and AFS-negative specimens. AFS: acid fast stain; + : positive; − : negative. N: sample size.

	Culture +	Culture −
**All specimens (N = 134)**		
HS-RTP +	77	11
HS-RTP −	2	44
N-RTP +	75	6
N-RTP −	4	49
**Pulmonary specimens (N = 73)**		
HS-RTP +	52	5
HS-RTP −	2	14
N-RTP +	52	2
N-RTP −	2	17
**Extra-pulmonary specimens (N = 61)**		
HS-RTP +	25	6
HS-RTP −	0	30
N-RTP +	23	4
N-RTP −	2	32
**On AFS positive specimens (N = 51)**		
HS-RTP +	45	5
HS-RTP −	1	0
N-RTP +	45	2
N-RTP −	1	3
**On AFS negative specimens (N = 83)**		
HS-RTP +	32	6
HS-RTP −	1	44
N-RTP +	30	4
N-RTP −	3	46

### Diagnostic accuracy of the N-RTP and HS-RTP assays

With respect to culture as the gold standard, the overall clinical sensitivity and specificity were 97.5% (77/79) versus 94.9% (75/59) and 80% (44/55) versus 89.1% (49/55) in the HS-RTP and N-RTP assays, respectively (*P* > 0.05). No obvious differences were observed between the HS-RTP and N-RTP assays regarding the sensitivity for pulmonary specimens (96.3%, 52/54) and AFS-positive specimens (97.8%, 45/46), while specificity was increased from 73.7% (14/19) to 87.5% (17/19) in the HS-RTP assay and from 0% (0/5) to 60% (3/5) in the N-RTP assay. The clinical sensitivity and specificity were 100% (25/25) versus 92.0% (23/25) and 83.3% (30/36) versus 88.9% (32/36) in the HS-RTP and N-RTP assays, respectively, for extrapulmonary specimens. Among AFS-negative specimens, the sensitivity and specificity were 97.0% (32/33) versus 88% (30/33) and 90.5% (44/50) and 92% (46/50) in HS-RTP and N-RTP, respectively. Moreover, the sensitivity and specificity of HS-RTP and N-RTP determined under various conditions in this study with the 95% confidence intervals are summarized in Table 4.

**Table 4:** Performance summary of HS-RTP, N-RTP, and CTM under various conditions, including on pulmonary specimens, on extrapulmonary specimens and on AFS-negative specimens. 95% CI: 95% confidence interval.

	Resolved performance (%) [95% CI]	
	Sensitivity	Specificity
**Overall specimens**		
HS-RTP	97.5 [91.2–99.3]	80.0 [67.6–88.5]
N-RTP	94.9 [87.7–98.0]	89.1[78.2–94.9]
CTM	81.8 [71.6–92.0]	98.7 [96.2–100.0]
**Pulmonary specimens**		
HS-RTP	96.3 [87.5–99.0]	73.7 [51.2–88.2]
N-RTP	96.3 [87.5–99.0]	89.5 [68.6–97.1]
CTM	86.1 [74.8–97.4]	100.0 [100.0–100.0]
**Extra-pulmonary specimens**		
HS-RTP	100.0 [86.7–100]	83.3 [68.1–92.1]
N-RTP	92.0 [75.0–97.8]	88.9 [74.5–95.6]
CTM	73.7 [53.9–93.4]	98.1 [94.6–100.0]
**AFS positive specimens**		
HS-RTP	97.8 [88.7–99.6]	0.0 [0.0–43.5]
N-RTP	97.8 [88.7–99.6]	60.0 [23.1–88.2]
CTM	100.0 [100.0–100.0]	20.0 [0.0–55.1]
**AFS negative specimens**		
HS-RTP	97.0 [84.7–99.5]	88.0 [76.2–94.4]
N-RTP	90.9 [76.4–96.9]	92.0 [81.2–96.9]
CTM	88.0 [79.0–97.0]	100.0 [100.0–100.0]

### Speed and costs

This study evaluated two molecular methods, N-RTP and HS-RTP, for the diagnosis of MTB. In terms of expense the N-RTP assay costs more than HS-RTP because it requires more materials, including an external primer, a high concentration of DNA polymerase, and abundant dNTPs. Taking into consideration the cost of the real-time PCR machine, the running cost of the N-RTP assay was approximately NT$25, representing an increase of 20% over the HS-RTP assay. In addition, N-RTP involves two PCR runs, and the first PCR step represents an additional step and, thus, additional time compared with HS-RTP. In conclusion, the HS-RTP assay is rapid and inexpensive, and the N-RTP assay is more expensive, time-consuming and precise.

## Discussion

In our previous study, an in-house IS6110-based qPCR assay (IS4) for the diagnosis of MTBC was developed. Compared to the commercially available Roche Cobas TaqMan MTB (CTM) qPCR assay, the results show high sensitivity in both pulmonary and extrapulmonary specimens, and the performance is not compromised under AFS-negative conditions; however, the specificity of the IS4 assay is much lower than that of CTM^35^. To improve the specificity of the IS4 assay, a single-tube nested-PCR assay (N-RTP) and a high-sensitivity real-time PCR assay (HS-RTP) were developed in this study by pairing IS4 and Ld2 and obtaining a new primer/probe set for the amplification of Lambda DNA as an internal control for MTBC detection. In addition, we designed a new external primer set (Nes6110) for the N-RTP assay. The performance of HS-RTP and N-RTP was evaluated in 134 clinical specimens and in comparison with mycobacterium culture results. The results indicated that the specificity in N-RTP was obviously increased in the different groups of specimens tested compared with HS-RTP, which is consistent with our expectations. However, we also noted that the sensitivity of N-RTP was slightly decreased in extrapulmonary and AFS-negative specimens, although this difference was not significant (*P* > 0.05). One possibility is that the Nes6110 primer was affected by inhibitors that were included in the extrapulmonary specimens or AFS-negative specimens.

Currently, there are two main testing platforms used in clinical examinations, for screening and confirmation, which are usually used for detecting drug abuse and conducting toxicology testing. Screening, which is also referred to as presumptive testing, is usually cost effective, rapid, and yields fast results; however, it is not very precise, and there are likely to be more false test results due to lower sensitivity and specificity. In contrast, confirmation testing requires greater time and expense than screening but usually provides definitive results. A similar concept was employed in this study. In the HS-RTP assay, which is cheaper, faster and shows high sensitivity (97.5%), the overall specificity is only 80%. In contrast, the N-RTP assay provides a more precise result and shows an obvious increase in specificity to 89.1%, but it is time-consuming and costly. On the basis of comprehensive consideration, we propose screening for suspected MTBC cases by first conducting the HS-RTP assay and then carrying out confirmation with the N-RTP assay. Two-stage nucleic acid amplification testing for screening and confirmation by HS-RTP and N-RTP in the diagnosis of MTBC achieved higher sensitivity and specificity.

Many studies have shown that the detection of MTBC by applying PCR to different genes or a combination of genes achieves higher sensitivity and specificity. IS1081 is a highly conserved gene in MTBC. It consists of four to six copies in the MTBC genome, even in *M. bovis* (which only contains a single copy of IS6110). As the number of copies between different MTBC members remains stable, several studies have reported the use of IS1081 as the NAAT target. It has been reported that IS1081-PCR sensitivity is increased compared with the use of rpoB, which encodes the β-subunit of bacterial RNA polymerase in MTBC, as a target in pleural fluid specimens^37^. IS1081 has also been combined with 23S rDNA and IS6110 to develop a single-tube triplex PCR assay, and the results indicated that this approach increased the differentiation between MTC and NTM^38^. *Khosravi *et al. demonstrated that among five target genes (IS1081, IS6110, hsk65kd, mbp64 and mtp40), only IS1081-based PCR showed an identical positivity rate (30.8%) to the results of AFS staining in pleural fluid specimens, and this strategy showed a significantly improved positivity rate in bone and wound specimens (33.3%)^39^. In addition, Fatolahzadeh et al. successfully used IS1081–PCR for the detection of pulmonary tuberculosis, and 78.2% of their isolates yielded positive results^40^. The sensitivity of IS1081-PCR has been reported to be higher than that of IS6110-PCR^41^; thus, it is a realistic screening method for the rapid identification of positive MTBC cases; however, it lacks the sensitivity of single copy-based strategies^42^. Ultra is a commercialized kit that targets IS6110, and IS1081 for detection and has been recommended by the WHO as a replacement for the Xpert cartridge (targeting only IS6110). Many reports have shown that the Ultra assay might exhibit higher sensitivity than Xpert; however, the WHO reported that the specificity of the Ultra assay is lower than that of the Xpert assay, and the cost is higher, which means that it will yield a higher proportion of false-positive results^43^. In summary, IS1081 may be a good target for the detection of MTBC in both pulmonary and extrapulmonary specimens, but the variable sensitivity of this approach in specimens of different origins has raised concern. In this study, a single-tube multiplex real-time PCR assay using IS6110/IS1081 as the molecular target was developed to identify MTBC in suspected cases. A comparison of partial IS1081 sequences among the MTBC members is shown in supplemental Fig. 1. Two and one single-nucleotide polymorphisms occurred in *M. africanum* and *M. canettii*, respectively. The IS1081 DNA sequence shares 99.9% identity with MTBC, which is similar to the result for the IS6110 sequence and meets our expectations. In addition, we designed a new outer primer (Nes1081) and inner primer/probe set (IS1081F, IS1081R and IS1081P) for IS1081 nested PCR in this study. To verify the performance of IS1081 nested PCR amplification, the sensitivity of the primer and probe set was investigated using the method described previously. The IS1081 amplicon was used to design a 6-carboxyl-X-rhodamine (ROX)-labeled IS1081P probe emitting red fluorescence (610 nm). The comparison of HS1081 and N1081 shows that the fluorescence of N1081 is slightly higher than that of HS1081 (Supplemental Fig. 2a, 2b). The primer efficiency of qPCR was 102.8% and 105.8% in the HS1081 and N1081 assays, respectively, and the overall results show a similar correlation coefficient between HS1081 and N1081 (Supplemental Fig. 2c). Compared with HS1081 (Ct values from 23.9 to 36.4), the N1081 results produced amplification curves with relatively lower Ct values (15.3 to 27.9), indicating that effective amplification occurred in the N1081 assay (Supplemental Fig. 2d), which may increase its sensitivity. The LOD of N1081 was estimated to be lower than 10 copies (Supplemental Table 2). On the basis of other reports, we assumed that the use of IS1081 may increase the sensitivity and specificity of the assay when combined with IS6110 for the detection of MTBC. However, an unexpected result was that among the 79 culture-positive specimens, only one specimen (from pleural fluid) yielded a positive result in N1081 and not in N-RTP. However, six specimens were N-RTP positive, while 2 pulmonary specimens and 3 extrapulmonary specimens were N1081 negative. One possibility is that the number of copies of IS6110 is higher than that of IS1081 in most patient specimens, and very few specimens show exactly opposite results. To our knowledge, *M. bovis* is the only species in this group in which the copy number of IS1081 is higher than that of IS6110 in the genome. However, among 3,321 patient isolates from Taiwan, 3,306 (99.5%) were found to be *M. tuberculosis*, and only 15 (0.5%) were *M. bovis*, and this pattern was especially prevalent in aborigines^44^. This result suggests that the prevalence of *M. bovis* is very low and may explain why we were unable to obtain significant positive results in terms of increased sensitivity and specificity in this study.

There are several limitations of this study that affect future applications. First, the sample size was not sufficient to reach statistical significance; thus, more specimens are required to investigate the diagnostic performance of the developed assays. In addition, we focused on developing IS61110 and IS1081 primer/probe sets, and many other genes that are suitable for NAATs should be compared with this assay in the future. Moreover, other NAAT methods have been used to diagnose TB, such as loop-mediated isothermal amplification (LAMP)^45^ and rolling circle amplification (RCA)^46^; these methods both exhibit high sensitivity and specificity and can be used for comparisons, including comparisons of speed and cost. Moreover, NAAT cannot discriminate the viability of MTBC pathogens. Thus, disease activity of MTBC cannot be determined in which other information, including AFS, mycobacterium culture, and clinical conditions are still necessary for accurate diagnosis and management of MTBC infection.

## Conclusion

The two-step nucleic acid amplification test provides high sensitivity and high specificity and thus can be used for screening and confirmation of MTBC. The performance of the test do not compromised in extra-pulmonary or AFS-negative specimens.

## Supplementary Information

Supplementary Information 1.

Supplementary Information 2.
