# Supplementary material for: Development of a two-step nucleic acid amplification test for accurate diagnosis of the Mycobacterium tuberculosis complex
Source: Sci Rep. 2021 Mar 11;11:5750. doi: 10.1038/s41598-021-85160-2 (PMC7952592; doi:10.1038/s41598-021-85160-2)
Supplement: Supplementary file 1 — Supplementary Information 1. [file 41598_2021_85160_MOESM1_ESM.docx]

**Development of a two-step nucleic acid amplification test for accurate diagnosis of the *Mycobacterium tuberculosis* complex**

Chien-Ru Lin ^1†^, Hsin-Yao Wang ^2, 3†^, Ting-Wei Lin^2^, Jang-Jih Lu^6, 7^, Jason Chia-Hsun Hsieh^4, 5^, Min-Hsien Wu^1,4, 8*^

^1^Graduate Institute of Biomedical Engineering, Chang Gung University, Taoyuan, Taiwan

^2^Department of Laboratory Medicine, Chang Gung Memorial Hospital at Linkou, Taoyuan City, Taiwan

^3^Ph.D. Program in Biomedical Engineering, Chang Gung University, Taoyuan City, Taiwan

^4^Division of Haematology/Oncology, Department of Internal Medicine, Chang Gung Memorial Hospital at Linkou, Taoyuan City, Taiwan

^5^Division of Haematology/Oncology, Department of Internal Medicine, New Taipei Municipal Hospital, New Taipei City, Taiwan

^6^School of Medicine, Chang Gung University, Taoyuan City, Taiwan

^7^Department of Medical Biotechnology and Laboratory Science, Chang Gung University, Taoyuan City, Taiwan

^8^Department of Chemical Engineering, Ming Chi University of Technology, New Taipei City, Taiwan

***Corresponding author**: Min-Hsien Wu, Ph.D.

Tel.: +886-3-2118800 ext 3599

Fax: +886-3-2118668

E-mail: mhwu@mail.cgu.edu.tw

† Lin and Wang contributed equally to this manuscript

**Keywords**: Nested PCR; *Mycobacterium tuberculosis*

### Supplementary Information


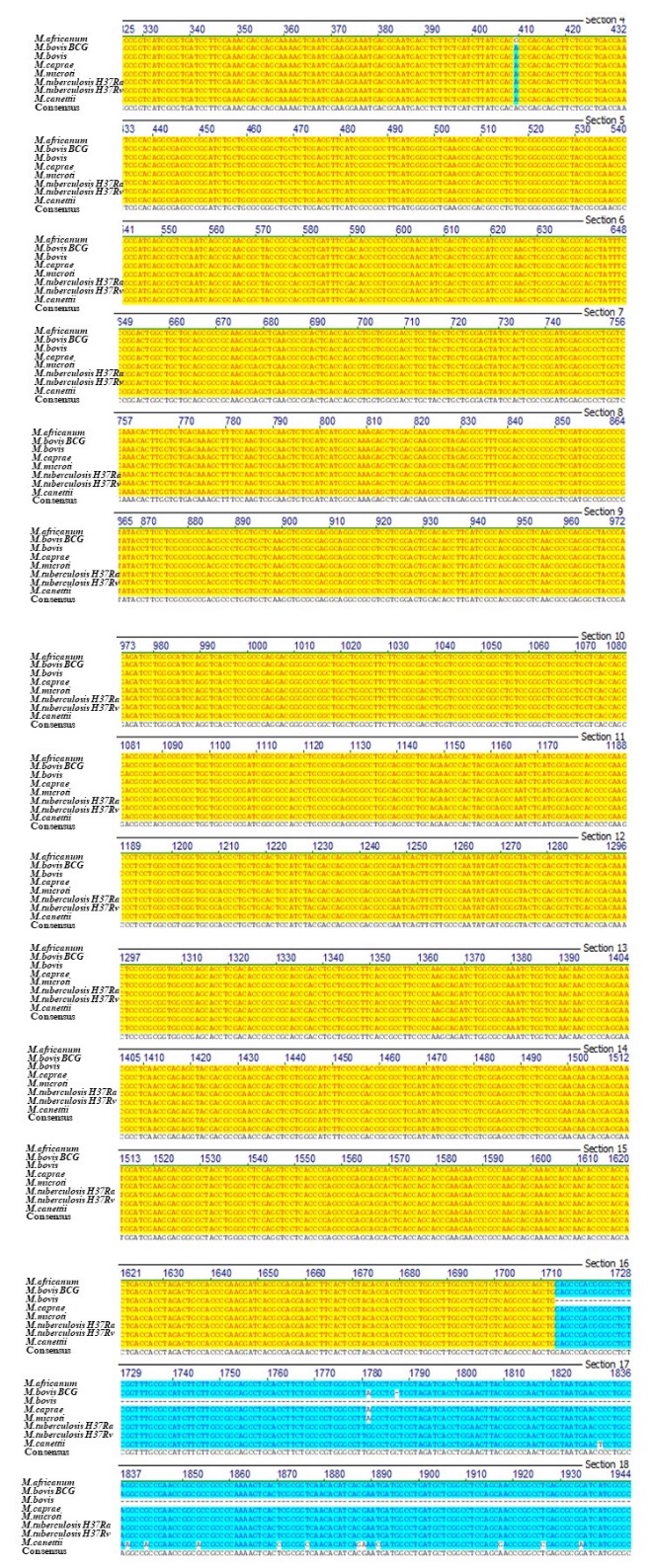


### Supplemental Figure 1. IS1081 sequence comparison among MTB complex.

Identical nucleotides are shown as red letters on a yellow background, and similar nucleotides are indicated as dark blue letters on a light blue background.

**
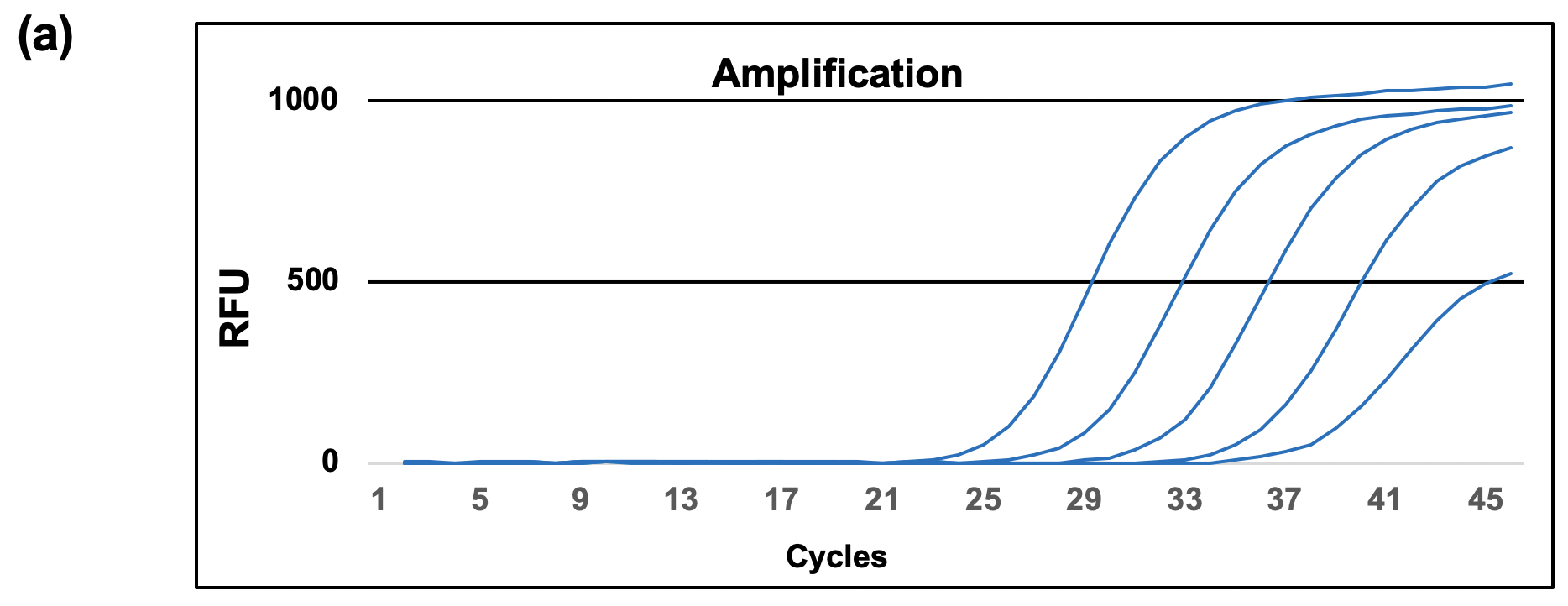
**

**
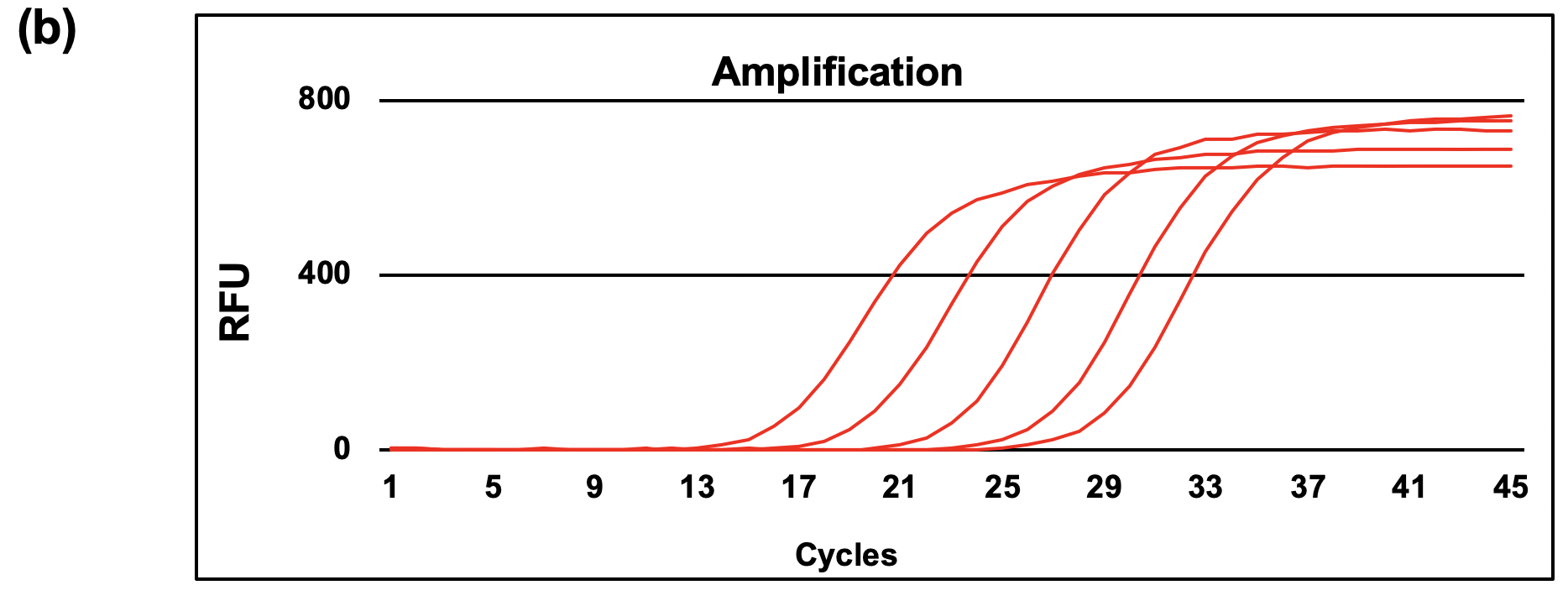
**

**
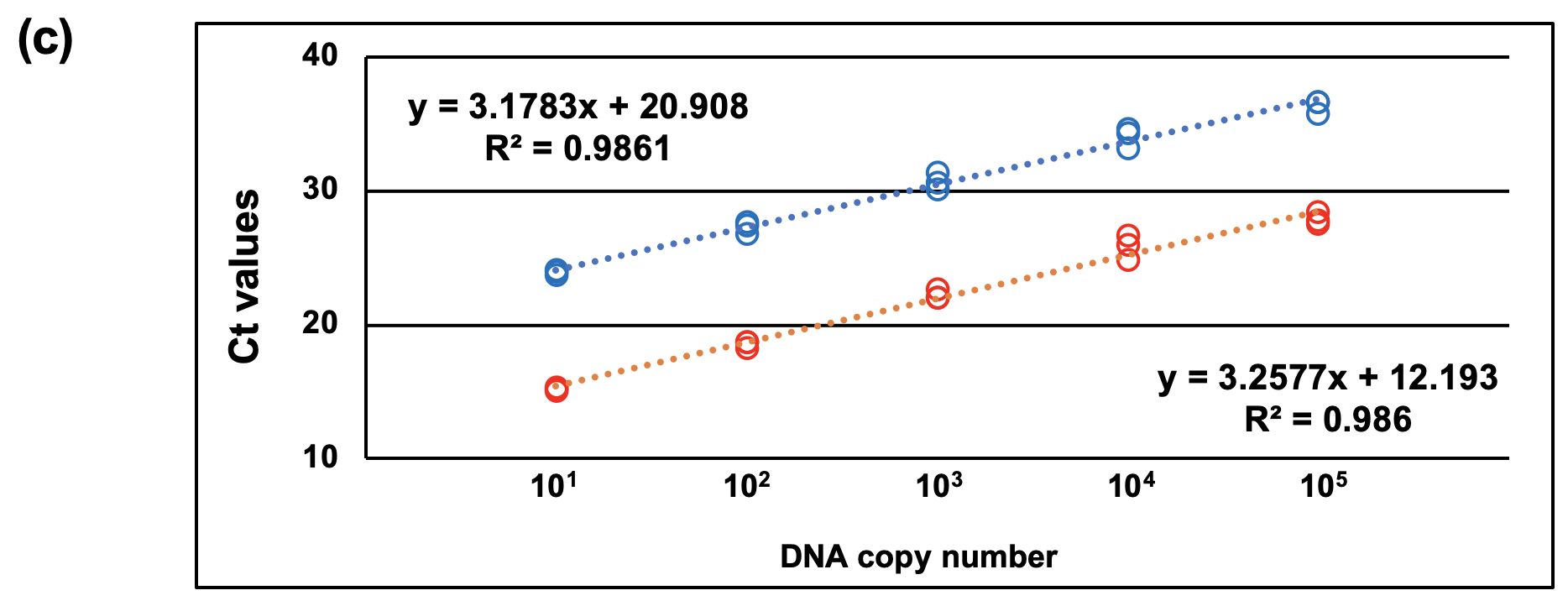
**

**
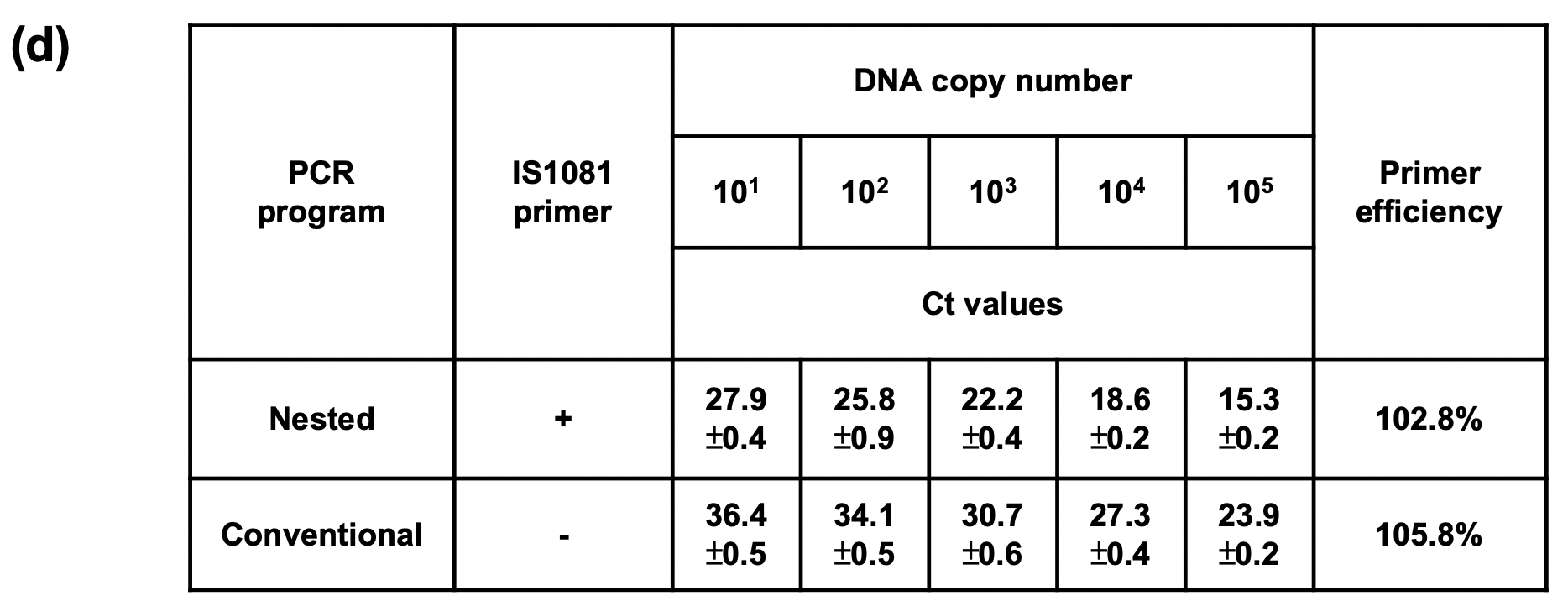
**

**Supplemental Figure 2. Single-tube nested real-time PCR assay using five samples of which Lambda DNA (1000 copies) was mixed with 10-fold serial dilutions of *IS1081*-containing synthetic vectors from 10^5^-10^1^ DNA copies.** Amplification curve of conventional real-time PCR **(a)** and single-tube nested real-time PCR **(b)**. Detection of *IS1081* target sequence with a FAM-labeled double quenched probe (495–520 nm) showing the increasing number of cycles required to detect reducing DNA copies. The relative fluorescence units (y axis) of the reaction is plotted against the Ct values (x axis). The amplification curves are representatives of three independent experiments. **(c)** Standard curve of single-tube nested real-time PCR (red circles) and conventional PCR (blue circles). The Ct values (y axis) were plotted against the log of the starting quantity of *IS1081*-containing synthetic vectors DNA copies (x axis) for each dilution. There are representatives of three independent experiments in each dilution. The slope, Ct values, Y-intercept, and R^2^ are shown. **(d)** The Ct values and primer efficiency with and without nested PCR are shown.
