# Supplementary material for: Development of a two-step nucleic acid amplification test for accurate diagnosis of the Mycobacterium tuberculosis complex
Source: Sci Rep. 2021 Mar 11;11:5750. doi: 10.1038/s41598-021-85160-2 (PMC7952592; doi:10.1038/s41598-021-85160-2)
Supplement: Supplementary file 2 — Supplementary Information 2. [file 41598_2021_85160_MOESM2_ESM.docx]

**Development of a two-step nucleic acid amplification test for accurate diagnosis of the *Mycobacterium tuberculosis* complex**

Chien-Ru Lin ^1†^, Hsin-Yao Wang ^2, 3†^, Ting-Wei Lin^2^, Jang-Jih Lu^6, 7^, Jason Chia-Hsun Hsieh^4, 5^, Min-Hsien Wu^1,4, 8*^

^1^Graduate Institute of Biomedical Engineering, Chang Gung University, Taoyuan, Taiwan

^2^Department of Laboratory Medicine, Chang Gung Memorial Hospital at Linkou, Taoyuan City, Taiwan

^3^Ph.D. Program in Biomedical Engineering, Chang Gung University, Taoyuan City, Taiwan

^4^Division of Haematology/Oncology, Department of Internal Medicine, Chang Gung Memorial Hospital at Linkou, Taoyuan City, Taiwan

^5^Division of Haematology/Oncology, Department of Internal Medicine, New Taipei Municipal Hospital, New Taipei City, Taiwan

^6^School of Medicine, Chang Gung University, Taoyuan City, Taiwan

^7^Department of Medical Biotechnology and Laboratory Science, Chang Gung University, Taoyuan City, Taiwan

^8^Department of Chemical Engineering, Ming Chi University of Technology, New Taipei City, Taiwan

***Corresponding author**: Min-Hsien Wu, Ph.D.

Tel.: +886-3-2118800 ext 3599

Fax: +886-3-2118668

E-mail: mhwu@mail.cgu.edu.tw

† Lin and Wang contributed equally to this manuscript

**Keywords**: Nested PCR; *Mycobacterium tuberculosis*

### Supplementary Information

**Supplemental Table 1. Strains used for specificity testing**

| Group | Species | N |
| --- | --- | --- |
| **NTM** | ***M. abscessus*** | **3** |
|  | ***M. avium*** | **1** |
|  | ***M. chimaera-intracellulare* group** | **1** |
|  | ***M. cosmeticum*** | **1** |
|  | ***M. farcinogene senegalense* group** | **1** |
|  | ***M. fortuitum*** | **1** |
|  | ***M. gordonae*** | **1** |
|  | ***M. mageriitum*** | **1** |
|  | ***M. peregrinum*** | **1** |
|  | ***M. kansasii*** | **1** |
|  | ***M. szulgai*** | **1** |
| **Bacteria** | ***A.baumannii*** | **2** |
|  | ***B-Strepto.Gr.B*** | **1** |
|  | ***E.coli*** | **1** |
|  | ***K. pneumoniae*** | **1** |
|  | ***P. aeruginosa*** | **1** |
|  | ***S. epidermidis*** | **1** |
| **Fungus** | ***A. flavus*** | **1** |
|  | ***A. niger*** | **2** |
|  | ***C. albicans*** | **1** |
|  | ***C. guilliermondii*** | **1** |
|  | ***C. krusei*** | **1** |
|  | ***C. lusitaniae*** | **1** |
|  | ***C. tropicalis*** | **1** |
|  | ***S. apiospermum*** | **1** |
|  | ***T. asahii*** | **1** |

Various non-tuberculous mycobacterial (NTM) strains, bacterial and fungus species used in the determination of the specificity HS-RTP and N-RTP assays. All tested negative by both assay. N means different batch of sample preparation and test.

**Supplemental Table 2. Limit of detection of IS1081-qPCR assay**

| DNA  copy number | N-RTP | | HS-RTP | |
| --- | --- | --- | --- | --- |
|  | **Average**  **Ct values**  **(**[**±**](http://mepopedia.com/?page=394)**SD)** | **Interpretation**  **(representation)** | **Average**  **Ct values**  **(**[**±**](http://mepopedia.com/?page=394)**SD)** | **Interpretation**  **(representation)** |
| **5** | **29.0**  **±0.3/ND** | **Positive**  **(16/20)** | **37.8**  **±0.6/ND** | **Positive**  **(19/20)** |
| **1** | **33.2**  **±0.5/ND** | **Positive**  **(12/20)** | **40.2**  **±0.7/ND** | **Positive**  **(9/20)** |
| **0** | **ND** | **Negative** | **ND** | **Negative** |
